# SEROTONIN AND DOPAMINE CIRCUITS MODULATE AGING IN RESPONSE TO FOOD CUES AND AVAILABILITY

**DOI:** 10.1093/geroni/igad104.3001

**Published:** 2023-12-21

**Authors:** Scott Leiser

**Affiliations:** University of Michigan, Ann Arbor, Michigan, United States

## Abstract

An organism’s ability to perceive and respond to changes in its environment is crucial for its health and survival. Multiple studies have shown that modifying this perception through genetic or environmental alterations can extend or shorten lifespan cell non autonomously. These results provide the rationale for identifying “longevity circuits” that could be used to extend healthspan and lifespan by using these signaling pathways. Here we reveal how the most well-studied longevity intervention, dietary restriction, acts in-part through a cell non-autonomous signaling pathway that is inhibited by the presence of multiple food cues. Using an intestinal reporter for a key gene induced by dietary restriction but suppressed by attractive food cues, we identify three compounds that block food cue effects in C. elegans, thereby increasing longevity as dietary restriction mimetics. These compounds and our additional data clearly implicate serotonin and dopamine circuits in limiting lifespan in response to food cues. We further identify multiple neurons, receptors and downstream signals within these circuits. Aspects of these pathways are conserved in D. melanogaster, suggesting at least some “longevity circuits” are conserved. Together, our results show that modulating food cue signaling through serotonin or dopamine circuits is a plausible approach to mimic the benefits of dietary restriction.

